# Reevaluating the Role of Megalin in Renal Vitamin D Homeostasis Using a Human Cell-Derived Microphysiological System

**DOI:** 10.14573/altex.1803161

**Published:** 2018-07-08

**Authors:** Brian D. Chapron, Alenka Chapron, Brian Phillips, Miracle C. Okoli, Danny D. Shen, Edward J. Kelly, Jonathan Himmelfarb, Kenneth E. Thummel

**Affiliations:** 1 Department of Pharmaceutics, University of Washington, Seattle, WA, USA; 2 Department of Medicine, University of Washington, Seattle, WA, USA

## Abstract

The role of megalin in the regulation of renal vitamin D homeostasis has previously been evaluated in megalin-knockout mice and rat proximal tubule epithelial cells. We revisited these hypotheses that were previously tested solely in rodent models, this time using a 3-dimensional proximal tubule microphysiological system incorporating primary human proximal tubule epithelial cells. Using this human cell-derived model, we confirmed that 25OHD_3_ is transported into the human proximal tubule epithelium via megalin-mediated endocytosis while bound to vitamin D binding protein. Building upon these findings, we then evaluated the role of megalin in modulating the cellular uptake and biological activity of 1α,25(OH)_2_D_3_. Inhibition of megalin function decreased the 1α,25(OH)_2_D_3_-mediated induction of both cytochrome P450 24A1 protein levels and 24-hydroxylation activity following perfusion with vitamin D binding protein and 1α,25(OH)_2_D_3_. The potential for reciprocal effects from 1α,25(OH)_2_D_3_ on megalin expression were also tested. Contrary to previously published observations from rat proximal tubule epithelial cells, 1α,25(OH)_2_D_3_ did not induce megalin gene expression, thus highlighting the potential for meaningful interspecies differences in the homeostatic regulation of megalin in rodents and humans. These findings challenge a recently promoted hypothesis, predicated on the rodent cell data, that attempts to connect 1α,25(OH)_2_D_3_-mediated regulation of renal megalin expression and the pathology of chronic kidney disease in humans. In addition to providing specific insights related to the importance of renal megalin in vitamin D homeostasis, these results constitute a proof-of-concept that human-derived microphysiological systems are a suitable replacement for animal models for quantitative pharmacology and physiology research.

## 1 Introduction

In humans, conversion of 25OHD_3_ to its bioactive form, 1α,25(OH)_2_D_3_, occurs primarily in the renal proximal tubule. It is a tightly regulated process, controlled by a number of intracrine and endocrine feedback loops (Wang et al., 2015; Dusso et al., 2005; Maiti and Beckman, 2007; Perwad et al., 2007). When levels of calcium are low, parathyroid hormone, a potent inducer of renal cytochrome P450 27B1 (CYP27B1), is released from the parathyroid gland to increase production of 1α,25(OH)_2_D_3_ in the kidneys (Dusso et al., 2005; Brenza et al., 1998). When systemic concentrations of 1α,25(OH)_2_D_3_ are elevated, vitamin D receptor (VDR)-dependent induction of renal cytochrome P450 24A1 (*CYP24A1*), the 24-hydroxylase responsible for the metabolic inactivation of 1α,25(OH)_2_D_3_ and 25OHD_3_, acts to reduce 1α,25(OH)_2_D_3_ and maintain mineral homeostasis (Jones et al., 2012).

In order for the proximal tubule epithelial cells (PTECs) to sense and respond to systemic demands for more or less 1α,25(OH)_2_D_3_, both 1α,25(OH)_2_D_3_ and its metabolic precursor, 25OHD_3_, must gain intracellular access. Both 1α,25(OH)_2_D_3_ and 25OHD_3_ circulate tightly bound to vitamin D binding protein (DBP) (Bikle et al., 1985, 1986). As a result, passive permeability of the unbound hormone and prohormone alone would yield low unbound intracellular concentrations (Dusso et al., 2005). Nykjaer et al. (1999) conducted a series of experiments in megalin-knockout mice and reported that the major route by which 25OHD_3_ accesses murine PTECs is via megalin-mediated endocytosis of the DBP-bound prohormone from the glomerular ultrafiltrate. Around the same time as these knockout mouse studies were being conducted, Liu et al. (1998) reported that 1α,25(OH)_2_D_3_ induced megalin expression in immortalized rat PTECs.

Taken together, the separate findings of Nykjaer et al. (1999) and Liu et al. (1998) suggested a role for megalin in the physiological maintenance of vitamin D homeostasis, both in health and disease. If 1α,25(OH)_2_D_3_ promotes megalin expression, and if megalin is essential for the renal delivery of 25OHD_3_, then diminished renal 1α,25(OH)_2_D_3_ synthesis, such as in chronic kidney disease (CKD) (Bosworth and de Boer, 2013), would reduce renal access of 25OHD_3_ and result in further reductions in 1α,25(OH)_2_D_3_ synthesis and progressively deteriorating vitamin D status. Predicated solely on rat cell data, this hypothesis of positive feedback has been promoted in a number of reviews (Dusso et al., 2011; Dusso, 2011; Kim and Kim, 2014). However, no follow-up studies evaluating the relationship between 1α,25(OH)_2_D_3_ and megalin expression in a more human-relevant system have thus far been conducted to verify this hypothesis.

Given known interspecies differences in the functionality of megalin-associated proteins, such as cubilin, that support megalin-mediated uptake of DBP (Amsellem et al., 2010; Nielsen et al., 2016; Nykjaer et al., 2001), a move away from animal models to experimental human studies is critical. However, this transition has long been hindered by the lack of a feasible and ethical means of delivering and sampling from an isolated human proximal tubule *in vivo*. The recent development of a perfusion-based 3-dimensional proximal tubule microphysiological system (PT-MPS) that recapitulates the physiological functions of the renal proximal tubule now permits further exploration of the role of megalin in the regulation of systemic vitamin D homeostasis (Weber et al., 2016). Observations of fluid sheer stress promoting endocytosis and cellular polarization suggest an advantage to studying endocytotic processes in perfusion-based systems (Raghavan et al., 2014; Raghavan and Weisz, 2015). Indeed, retention of microvilli, the subcellular structures upon which functional (i.e., capable of binding extracellular ligands) megalin protein is localized, have been confirmed in the apical membranes of PTECs cultured in the PT-MPS (Weber et al., 2016; Sun et al., 2017). Importantly, the specific design of the PT-MPS permits the delivery of ligands of interest selectively to these apical cell surfaces, facilitating the functional characterization of megalin, a protein that is primarily localized to the apical membrane of PTECs (Kerjaschki et al., 1984).

Using the *in vivo*-like environment of the PT-MPS and conventional 2-dimensional human PTEC cultures, we tested the hypotheses that (1) megalin modulates the intracellular disposition of DBP-bound vitamin D metabolites, (2) megalin-mediated processes modulate the physiological activity of 1α,25(OH)_2_D_3_, and (3) megalin gene expression is a target for reciprocal regulation by 1α,25(OH)_2_D_3_ in the proximal tubule.

## 2 Materials and methods

### Chemicals and reagents

Bovine serum albumin, hydrocortisone, Tween-20, and Triton X-100 were purchased from Sigma-Aldrich (St. Louis, MO). D-Sucrose was obtained from Fisher Scientific (Itasca, IL). 16% Formaldehyde (methanol free) was purchased from Polysciences (Warrington, PA). Vitamin D metabolites were obtained from Toronto Research Chemicals (Toronto, Ontario). Dulbecco’s phosphate-buffered saline with (DPBS) and without (DPBS^++^) calcium and magnesium, 50:50 Dulbecco’s modified Eagle’s medium with Ham’s F-12 (DMEM/F12), Hank’s balanced salt solution, penicillin-streptomycin-amphotericin B, insulin-transferrin-selenium A solution (ITS-A), TRIzol^®^ reagent, High Capacity cDNA Reverse Transcription Kit with RNase Inhibitor, TaqMan gene expression assays, fetal bovine serum (FBS), trypsin ethylenediamine tetraacetic acid (EDTA), collagenase type IV, ProLong Gold^®^ Antifade reagent with/without 4’,6-diamidino-2-phenylindole (DAPI), and rabbit anti-human *CYP24A1* antibody were obtained from Thermo-Fisher (Waltham, MA). Mixed type human vitamin D binding protein (DBP) was purchased from Athens Research & Technology (Athens, GA). Alexa Fluor 594 conjugated donkey anti-mouse IgG, Alexa Fluor 488 conjugated donkey anti-rabbit IgG, Alexa Fluor 488 conjugated donkey anti-goat IgG, rabbit anti-megalin and mouse anti-sodium-potassium ATPase antibodies were purchased from Abcam (Cambridge, MA). Microfluidic platforms were obtained from Nortis (Woodinville, WA). Human receptor-associated protein (RAP) was purchased from Innovative Research (Novi, MI). Non-pepsinized rat tail collagen I was purchased from Ibidi (Martinsried, Germany). Collagen IV, Matrigel^®^, Transwell^®^ inserts and tissue culture-treated 6-well plates were obtained from Corning (Corning, NY).

### Cell culture

Healthy resections of human kidney cortical tissue were obtained during the surgical removal of renal cell carcinomas at the University of Washington Medical Center. The protocol was approved by the University of Washington Human Subjects Institutional Review Board (protocol # STUDY00001297). Human PTECs were isolated from kidney cortical tissue and cultured as previously described (Weber et al., 2016). Briefly, kidney cortical tissue was diced and subsequently incubated under agitation for 30 min at 37°C in a 1 mg/ml solution of collagenase type IV in Hank’s balanced salt solution. The free cell-containing supernatant was then transferred to new conical tubes and washed with PTEC culture medium (DMEM/F12 medium supplemented with insulin, transferrin, selenium, penicillin, streptomycin, amphotericin B, and 50 nM hydrocortisone). The cell suspension was centrifuged at 200 g for 5 min and the supernatant was aspirated. The cell pellet was then resuspended in PTEC culture medium and transferred to tissue culture flasks. Cell culture medium was replaced after 24 h and then every 3 d thereafter. Cells were expanded and subcultured in tissue culture treated flasks. Cell detachment was performed using 0.05% trypsin with EDTA and DMEM/F12 medium containing 10% FBS was used for enzymatic quenching. For use in experiments, PTECs (passage 1–5) were either seeded into well plates, Transwell^®^ inserts or single-channel Nortis microfluidic tubules to constitute a proximal tubule microphysiological system (PT-MPS). Cells cultured in all platforms were maintained under previously established “standard” cell culture conditions of 5% CO_2_, 37°C and PTEC culture medium (Weber et al., 2016). Cells cultured in the PT-MPS were perfused with medium at a rate of 0.5 μl/min.

### Statistical analysis

All statistical analyses were conducted using GraphPad Prism version 5.04 (GraphPad Software, La Jolla, CA). Data in figures depicts the mean ± SEM. All statistical tests evaluating “fold-changes” in the data (i.e., ratios) utilized log-transformed data to ensure parity between fold-reductions and fold-increases. Whenever possible, data was paired by kidney tissue donor. This paired comparison of log-transformed ratios is referred to throughout this paper as a ratio t-test. Statistically significant results are marked with *, p < 0.05 and **, p < 0.01.

### Megalin localization in cultured human PTECs

Because validated commercially-available antibodies for megalin require heat-mediated antigen retrieval, and the MPS platform currently does not permit this technique, we performed immunocytochemical staining for megalin protein localization in 2-dimensional Transwell^®^ cell culture inserts. Briefly, PTECs were seeded at a density of 2 × 10^5^ cells onto collagen IV-coated transparent Transwell^®^ inserts and allowed to attach for 5 h. Medium was then removed from the inserts and replaced with medium containing 0.25 mg/ml Matrigel^®^ and left overnight. The next day, the medium was replaced with medium without Matrigel^®^ and the cells were cultured further under standard conditions. After 7 days, the cells were fixed in a solution of 4% formaldehyde and 2% sucrose for 10 min. They were then incubated in 50 mM ammonium chloride for 30 min and rinsed 3 times with DPBS^++^. Next, cells were incubated in a solution of 0.05% Tween-20 in 10 mM sodium citrate buffer (pH = 6) for 20 min at 100°C. Cells were allowed to cool to room temperature (RT) before being blocked with PTB (a solution of 0.1% Triton X-100 and 5% bovine serum albumin in DPBS^++^) for 30 min. Rabbit anti-megalin and mouse anti-sodium-potassium ATPase primary antibodies in PTB were added to the inserts and incubated at RT for 30 min. Controls for non-specific binding of secondary antibodies were simultaneously incubated with PTB in the absence of primary antibodies. Information on primary antibodies is provided in Table S1^1^. The inserts were washed three times with DPBS^++^, and the cells were then incubated with a 1:1000 dilution of both Alexa Fluor 594 conjugated donkey anti-mouse IgG and Alexa Fluor 488 conjugated donkey anti-rabbit IgG for 30 min at RT. The cells were rinsed 3 times with DPBS^++^, and exposed to a 30-min incubation with a 1:3 dilution of 4’,6-diamidino-2-phenylindole (DAPI) in DPBS^++^. The cells were again rinsed 3 times with DPBS^++^ before the porous membrane of the cell culture insert was extracted using a scalpel and tweezers. The membrane was mounted in deionized water on glass microscope slides and imaged using a Zeiss LSM 780 confocal microscope (Carl Zeiss, Oberkochen, Germany). Confocal images were processed in Velocity software version 6.3 from PerkinElmer (Waltham, MA).

### Comparison of the effects of DBP and FBS on the 1α,25(OH)_2_D_3_-mediated regulation of CYP24A1 enzymatic activity in the PT-MPS

**Fig. 1:**
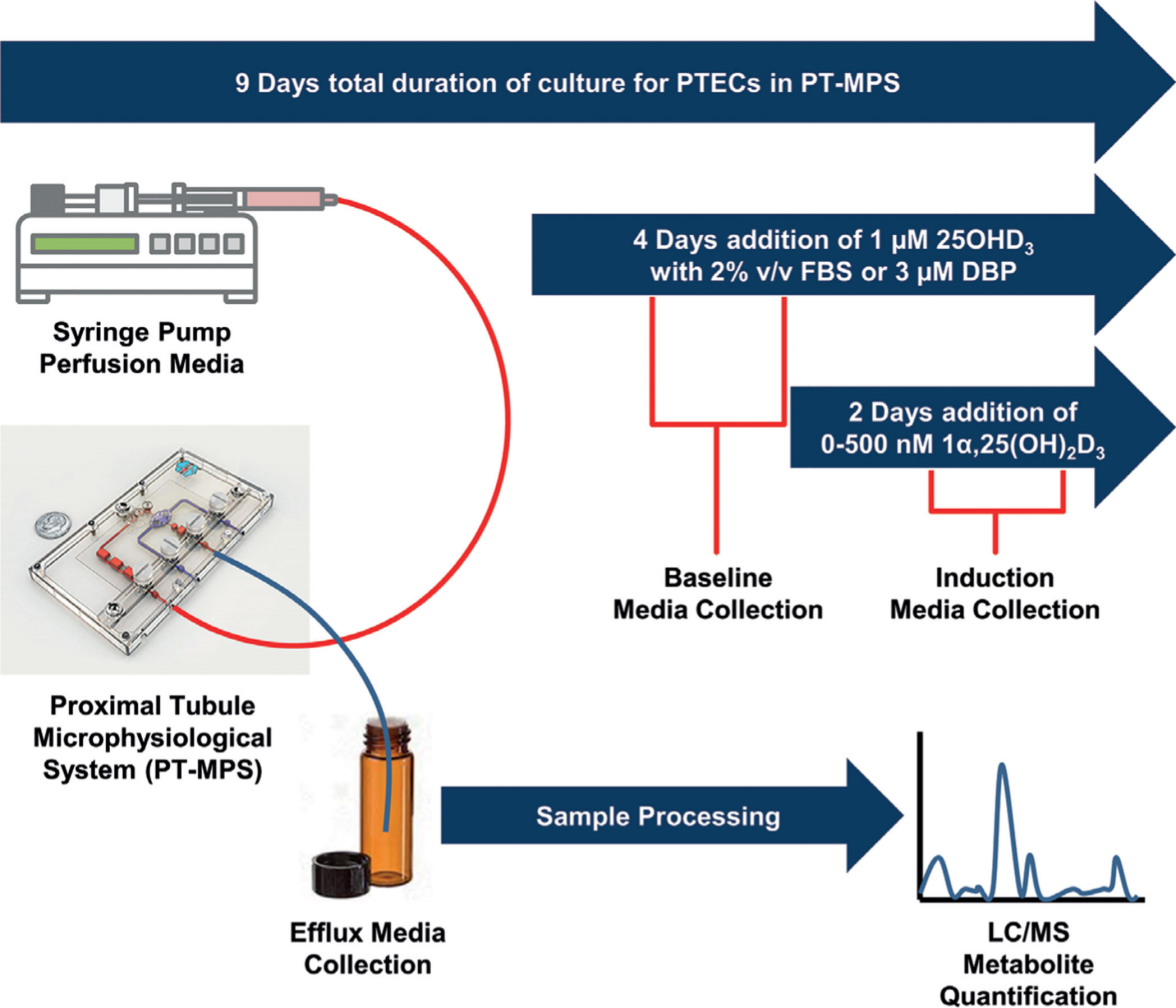
General scheme for experiments evaluating dose-dependent regulation of CYP24A1 by 1α,25(OH)_2_D_3_ in the PT-MPS PT-MPS are perfused with medium containing 1 μM 25OHD_3_ for 48 h with either 2% FBS or 3 μM DBP serving as a carrier vehicle for vitamin D metabolites. Baseline 24,25(OH)_2_D_3_ formation clearance is determined for each PTMPS from metabolite concentrations in the medium exiting the PT-MPS during the 24 to 48-h period of the “baseline” phase. The various PT-MPS then receive a range of 1α,25(OH)_2_D_3_ concentrations or vehicle control for the subsequent 48 h. The CYP24A1 substrate (1 μM 25OHD_3_) and the respective carrier protein source (FBS or DBP) for each PT-MPS is continued throughout the “induction” phase. The 24,25(OH)_2_D_3_ formation clearance during the “induction” phase is determined from the efflux medium of each PT-MPS during hours 24 to 48 of the 2-day collection interval following the initiation of 1α,25(OH)_2_D_3_ co-treatment. Fold-increase in 24,25(OH)_2_D_3_ formation clearance from the baseline to the induced state is calculated for each PT-MPS receiving 1α,25(OH)_2_D_3_ and standardized as a percentage increase in 24,25(OH)_2_D_3_ formation clearance over the vehicle control.

Having confirmed the suitability of purified human DBP as a delivery vehicle for vitamin D metabolites (See Fig. S1^1^), we conducted an exploratory experiment (outlined in Fig. 1) with a single donor comparing the relative effects of the two delivery vehicles (FBS and DBP) on the 1α,25(OH)_2_D_3_-mediated induction of 24-hydroxylation activity. Human PTECs from a single donor were cultured in the PT-MPS for 5 days post seeding, as described. All PT-MPS received 1 μM 25OHD_3_ for 48 h. Using Equation 1, baseline 24,25(OH)_2_D_3_ formation clearance (*CL*_*f*_) was determined for each PT-MPS from metabolite concentrations in the medium exiting the PT-MPS during hours 24 to 48 of the 2-day collection interval, following the addition of 25OHD_3_ to the perfusion medium.

(1) $$CL_{f}=\frac{Net\;Effluent\;Appearance\;Rate_{24,25{\left(OH\right)}_{2}D_{3}}}{{\left[25OHD_{3}\right]}_{outflow\;}}$$

In order to assess dose-dependency in the VDR-mediated induction of *CYP24A1* expression and activity, the 16 tubules were randomly assigned to groups of 4 to receive either 500, 100, 10 nM of the VDR ligand, 1α,25(OH)_2_D_3_, or 0.1% ethanol vehicle control for the subsequent 48 h. The *CYP24A1* substrate, 1 μM 25OHD_3_, was also continued in all PT-MPS throughout the treatment phase. The 24,25(OH)_2_D_3_ formation clearance during the “induction” phase was determined from medium exiting each PT-MPS during hours 24 to 48 of the 2-day collection interval following the initiation of 1α25(OH)_2_D_3_ induction. The experiment was conducted with either 2% FBS or 3 μM DBP serving as the carrier vehicle for vitamin D metabolites. Fold-increase in *CL*_*f*_ from the baseline (*CL*_*f*_ *(baseline phase,treatment)*) to the induced state (*CL*_*f*_ *(baseline phase,treatment)*) was calculated for each PT-MPS and standardized to the fold-change in *CL*_*f*_ from the baseline *CL*_*f (base*_*line phase,treatment)*) to the “induction” phase *CLf(baseline phase,treatment)*) of the vehicle control (Equation 2).

(2) $$\%\;Increase\;=\{\left(\frac{CL_{f(induction\;phase,treatment)}}{CL_{f(baseline\;phase,treatment)}}/\frac{CL_{f(induction\;phase,vehicle)}}{CL_{f(baseline\;phase,vehicle)}}\right)-1\}\times 100\%$$

The parameters of maximal induction (*E*_*max*_) and the concentration of 1α,25(OH)_2_D_3_ at which half maximal induction is observed (*EC*_*50*_) were then estimated using the simple *E*_*max*_ model, outlined in Equation 3.

(3) $$\%\;Increase\;=\frac{E_{max}\times \left[1\alpha 25{\left(\text{OH}\right)}_{2}D_{3}\right]}{EC_{50}+\left[1\alpha 25{\left(\text{OH}\right)}_{2}D_{3}\right]}$$

The estimated *E*_*max*_ and *EC*_*50*_ for the dose-dependent induction of 24-hydroxylation activity was then visually compared between PT-MPS supplemented with FBS *versus* DBP.

### Evaluation of the effects of megalin inhibition on the cellular uptake and 24-hydroxylation of 25OHD_3_ in the PT-MPS

PT-MPS were cultured under standard conditions (16–20 PT-MPS per donor) for 5 days. Then PT-MPS were perfused (0.5 μl/min) for 48 h with 3 μM DBP-supplemented medium containing 500 nM 1α,25(OH)_2_D_3_. This 48-h pre-incubation of 1α,25(OH)_2_D_3_ was employed to induce *CYP24A1* activity and enhance sensitivity to quantify the 24-hydroxylation of the relatively lower 25OHD_3_ concentrations. In order to remove residual 1α,25(OH)_2_D_3_ and promote the equilibration of incoming vitamin D metabolites, an 8 h accelerated perfusion (2.5 μl/min) of DBP-supplemented medium without 1α,25(OH)_2_D_3_, but containing different 25OHD_3_ concentrations (ranging from 0.25 to 3 μM), was administered to the PT-MPS. Perfusion with 25OHD_3_ in the DBP-supplemented medium was then reduced to 0.5 μl/min and a specific inhibitor of receptor-mediated (e.g., megalin-mediated) endocytosis (1 μM RAP – receptor associated protein) was administered to half of the PT-MPS at each 25OHD_3_ concentration (Niemeier et al., 1999; Rowling et al., 2006). Medium was collected for 1 day and concentrations of 25OHD_3_ and 24,25(OH)_2_D_3_ were determined using a previously established LC-MS/MS method (Wang et al., 2011; Weber et al., 2016). The disappearance rate of 25OHD_3_ from the perfusion medium was calculated according to Equation 4.

(4) $$25OHD_{3}\;Disappearance\;Rate\;=\frac{{\left[25OHD_{3}\right]}_{input\;}-{\left[25OHD_{3}\right]}_{out\;flow\;}}{\;Duration\;of\;collection\;Interval/Volume\;collected\;}$$

The net appearance rate for 24,25(OH)_2_D_3_ was also calculated across the range of 25OHD_3_ input concentrations (see Equation S1^1^). Given the roughly proportional increase in both 24,25(OH)_2_D_3_ net appearance and 25OHD_3_ disappearance rate across the range of 25OHD_3_ input concentrations, a simple linear regression model was fit to the data. Under these linear conditions, the slope of the 24,25(OH)_2_D_3_ net appearance and 25OHD_3_ disappearance rate across the concentration reflects the intrinsic clearance of 24,25(OH)_2_D_3_ appearance (*CL*_*int,24,25(OH)2D3*_) and 25OHD_3_ disappearance (*CL*_*int,24,25(OH)2D3*_), respectively. The effect of RAP on each intrinsic clearance was evaluated using ratio t-tests paired by kidney tissue donor (n = 5 biological replicates, all from male donors). Insufficient numbers of female donors yielding viable cells and the small sample size precluded an assessment of potential sex-dependent effects of RAP on the variables of interest. De-identified subject information for each kidney tissue donor is provided in Table S2^1^.

### Effect of megalin inhibition on 1α,25(OH)_2_D_3_-mediated induction of CYP24A1 enzymatic activity in the PT-MPS

The experimental design outlined in Figure 1 was modified so that all PT-MPS received 3 μM DBP as the carrier protein source. During the induction phase, half of the PT-MPS, per 1α,25(OH)_2_D_3_ concentration, additionally received 1 μM RAP. Fold induction of 24,25(OH)_2_D_3_ formation clearance was calculated as before and compared in the presence and absence of RAP. The experiment was then repeated for a total of 5 donors (4 males and 1 female), two of which were also evaluated in the presence of 2000 nM 1α,25(OH)_2_D_3_ to confirm that near-maximal induction was likely achieved where 500 nM 1α,25(OH)_2_D_3_ was the highest evaluated concentration. The shifts in *EC*_*50*_ and *E*_*max*_ were then evaluated using a ratio t-test comparing the estimated parameters from each donor in the presence and absence of RAP. Insufficient numbers of female donors yielding viable cells and the small sample size precluded an assessment of sex-dependent effects of RAP on the variables of interest. De-identified subject information for each kidney tissue donor is provided in Table S3^1^.

### Effect of megalin inhibition on 1α,25(OH)_2_D_3_-mediated induction of CYP24A1 protein accumulation in the PT-MPS

PT-MPS were maintained for 5 days under standard culture conditions. The culture medium was then supplemented with 3 μM human DBP and the cells were treated with either 500 nM 1α,25(OH)_2_D_3_ in the presence or absence of 1 μM RAP or 0.1% ethanol vehicle control. After 2 days of culture under these treatment conditions, the cells were fixed by flowing a solution of 4% formaldehyde and 2% D-sucrose in DPBS^++^ at 10 μl/min through the PT-MPS for 20 min. Using a previously established method (Weber et al., 2016), staining was conducted with a 1:200 dilution of goat anti-*CYP24A1* and a 1:1000 dilution of Alexa Fluor 488 conjugated donkey anti-goat IgG (secondary antibody). Information on primary antibodies is provided in Table S1^1^. Control PT-MPS tubules were perfused with secondary antibody in the absence of primary antibody pre-incubation in order to assess non-specific binding of the secondary antibody. Images of the PT-MPS tubules were captured on a Nikon Eclipse Ti fluorescent microscope (Melville, NY).

### Characterization of the effects of 1α,25(OH)_2_D_3_ on megalin gene expression

In order to increase total mRNA yield and improve sensitivity for megalin gene expression, human PTECs were cultured in collagen IV-coated 6-well plates, rather than the PT-MPS. The cells were cultured under standard conditions for 5 days before being exposed to a range of concentrations (0–500 nM) of 1α,25(OH)_2_D_3_. After 24 h exposure, the cells were homogenized in 1 ml of TRIzol^®^ reagent. The samples were collected and stored at −80°C until processing and analysis. The mRNA was then isolated according to the TRIzol^®^ manufacturer-supplied protocol and quantified on a NanoDrop ND-2000 spectrophotometer from Thermo-Fisher (Waltham, MA). Isolated RNA was mixed with other components of the high-capacity cDNA reverse transcription kit according to the manufacturer-supplied protocol. Reverse transcription was conducted in a PTC-200 thermal cycler (Bio-Rad, Hercules, CA) under the following conditions: 10 min at 25°C, 120 min at 37°C, and 5 sec at 85°C. Quantitative real-time polymerase chain reactions were then performed under the following conditions: warm up at 50°C for 10:00, followed by 40 cycles of 95°C for 5:10, and 60°C for 0:30. TaqMan gene expression assays were used to evaluate the dose-dependent effects of 1α,25(OH)_2_D_3_ on *megalin*, *CYP24A1* (inductive control) and *CYP27B1* (suppressive control) gene expression. The ΔΔCt method, using glyceraldehyde 3-phosphate dehydrogenase (*GAPDH*) as a housekeeping gene, was employed to quantify relative amounts of *megalin* (i.e., *LRP2*), *CYP24A1* and *CYP27B1* mRNA transcripts in all samples. A one-way ANOVA, treating different 1α,25(OH)_2_D_3_ doses in a given donor as repeated measures, was performed on the log-transformed data. Post-hoc ratio t-tests (n = 6 biological replicates) between each 1α,25(OH)_2_D_3_ dose and the vehicle control (0.1% ethanol) were then conducted for each of the genes evaluated (*megalin*, *CYP24A1* and *CYP27B1*). Levels of *CYP24A1* RNA were below the limit of quantification in the vehicle control group, so the group was omitted from the statistical analysis. Post-hoc t-tests for *CYP24A1* compared gene expression in cells treated with 1 nM 1α,25(OH)_2_D_3_ (rather than vehicle control) to those treated with higher doses.

## 3 Results

### 3.1 Megalin localization in cultured human PTECs

**Fig. 2:**
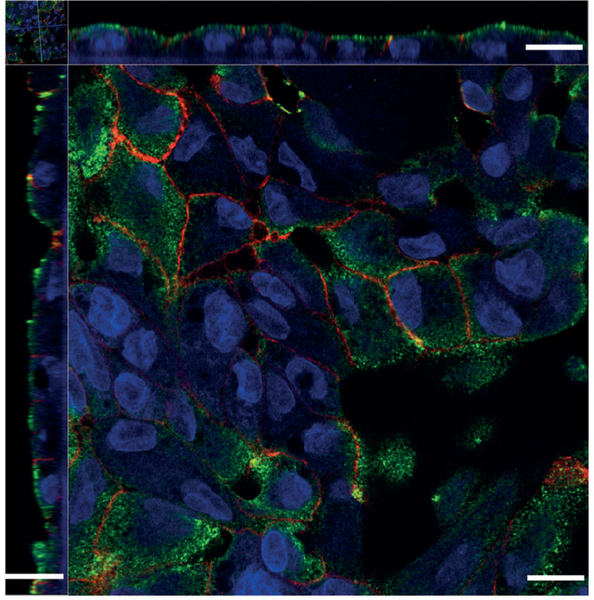
Megalin protein localization in PTECs Megalin (green signal) is preferentially expressed along the apical and adjacent subapical regions of cultured human proximal tubule epithelial cells (PTECs), as is seen *in vivo*. Sodium-potassium ATPase (red signal) was used as a counterstain for the cells’ basolateral membranes. Scale bar represents 10 μm.

Megalin was expressed along the apical and adjacent subapical regions of cultured human PTECs (Fig. 2). Sodium-potassium ATPase served as a counterstain for cell basolateral membranes (Molitoris et al., 1992; Secker et al., 2017). Proper expression and cellular localization of megalin confirmed the suitability of our *in vitro* system for studying megalin-mediated luminal reuptake of vitamin D metabolites.

### 3.2 DBP and FBS differentially affect the regulation of CYP24A1 activity by 1α,25(OH)_2_D_3_

**Fig. 3:**
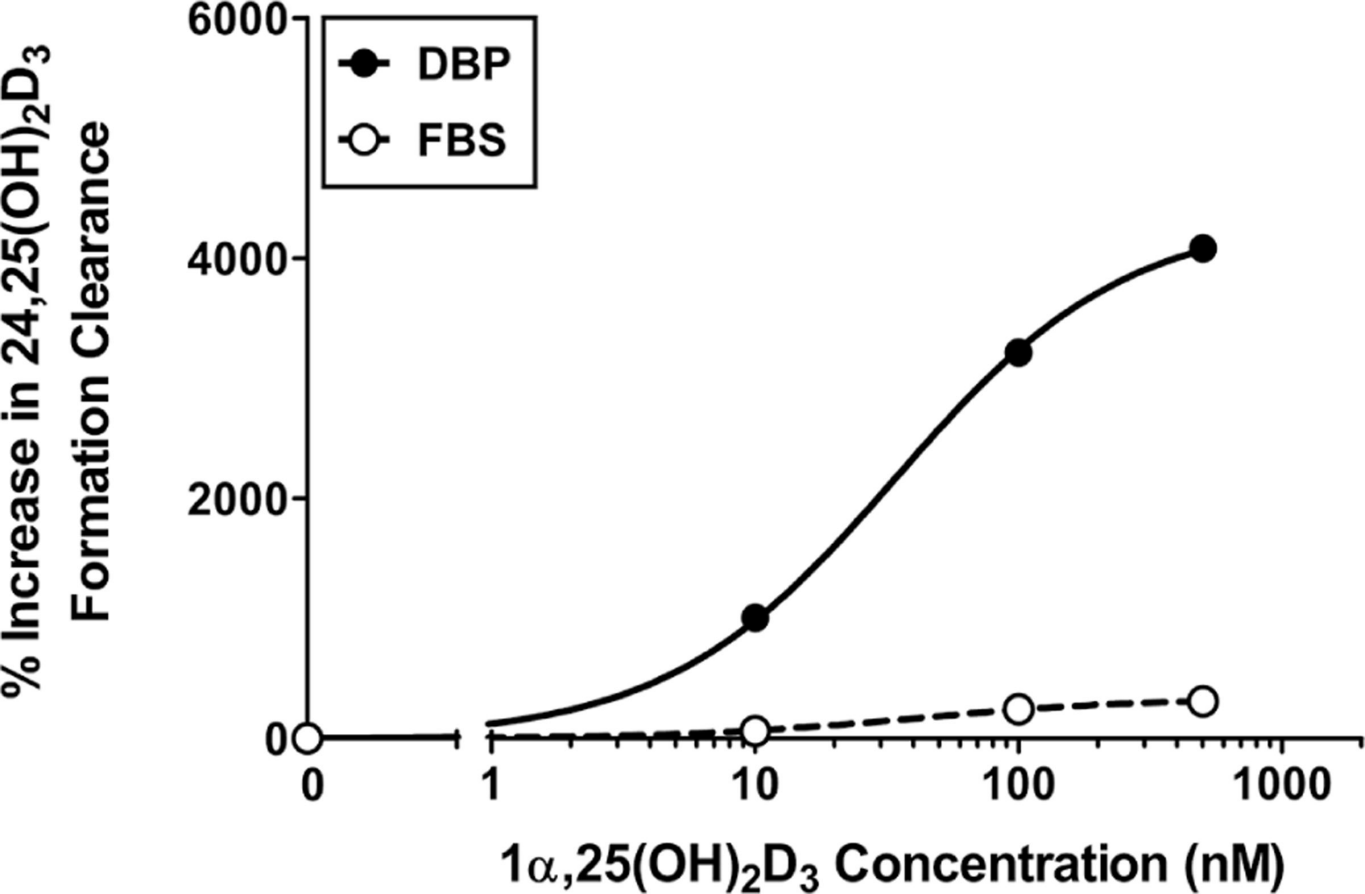
Relative effects of DBP and FBS on 1α,25(OH)_2_D_3_- mediated regulation of CYP24A1 activity in the PT-MPS Co-incubation of 25OHD_3_ and 1α,25(OH)_2_D_3_ across a range (0–500 nM) of concentrations resulted in a dose-dependent increase in the appearance of 24,25(OH)_2_D_3_ in the efflux of both DBP-and FBS-supplemented perfusion media. The maximal induction (*E*_*max*_) of 24,25(OH)_2_D_3_ formation clearance in DBP-supplemented medium was greater than that seen with FBS coincubation.

DBP is considered essential for megalin-mediated uptake of 25OHD_3_ (Nykjaer et al., 1999). Thus, the switch to purified human DBP from the previously established delivery vehicle, fetal bovine serum (FBS) (Weber et al., 2016), represents an improvement in facilitating a more controlled megalin-dependent delivery of vitamin D metabolites. In order to validate the use of DBP as a delivery vehicle, we first confirmed the successful uptake and metabolism of 25OHD_3_ to the PT-MPS in the presence of purified DBP (Fig. S1^1^). Next, we compared the relative effects of DBP and FBS on 1α,25(OH)_2_D_3_-mediated induction of *CYP24A1* activity in the PT-MPS. Co-administration of 25OHD_3_ and a range of 1α,25(OH)_2_D_3_ concentrations resulted in a dose-dependent increase in the appearance of 24,25(OH)_2_D_3_ in the efflux of both DBP-and FBS-supplemented perfusion media (Fig. 3). The maximal induction (*E*_*max*_) of 24,25(OH)_2_D_3_ formation clearance in DBP-supplemented medium was over 40-fold greater than the baseline activity and approximately 10-fold greater than the increase with FBS. Based on these results and given their physiological relevance, the remaining experiments were conducted using DBP for the delivery of vitamin D metabolites.

### 3.3 RAP inhibits cellular uptake of DBP-bound 25OHD_3_

**Fig. 4:**
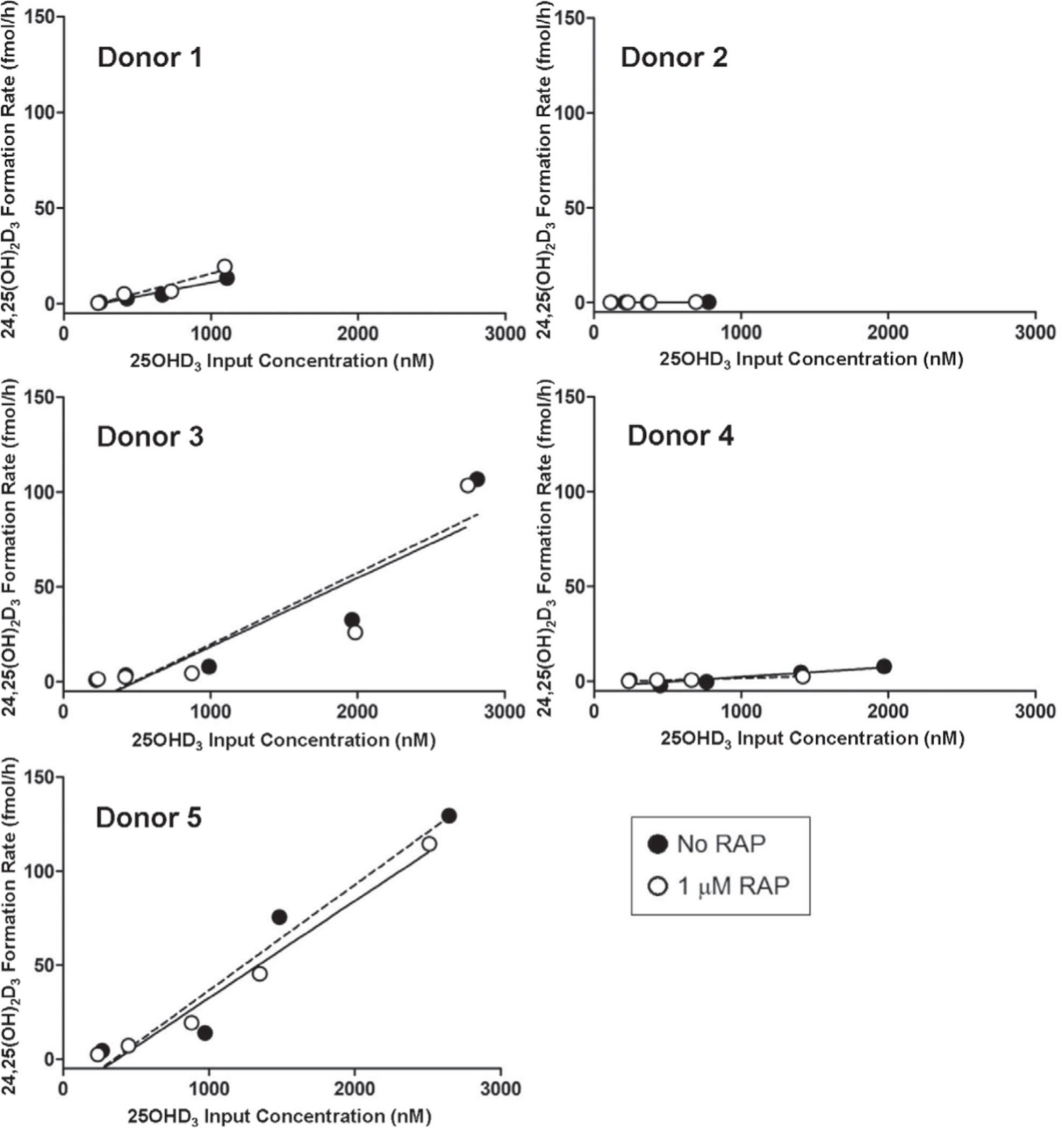
Megalin inhibition does not affect the formation of 24,25(OH)_2_D_3_ from 25OHD_3_ in the PT-MPS The rate of formation of 24,25(OH)_2_D_3_ from 25OHD_3_ was observed to be roughly proportional to the 25OHD_3_ input concentrations across the measured range of input concentrations tested. There was no significant effect of RAP on the estimated intrinsic formation clearance of 24,25(OH)_2_D_3_ from 25OHD_3_.

**Fig. 5:**
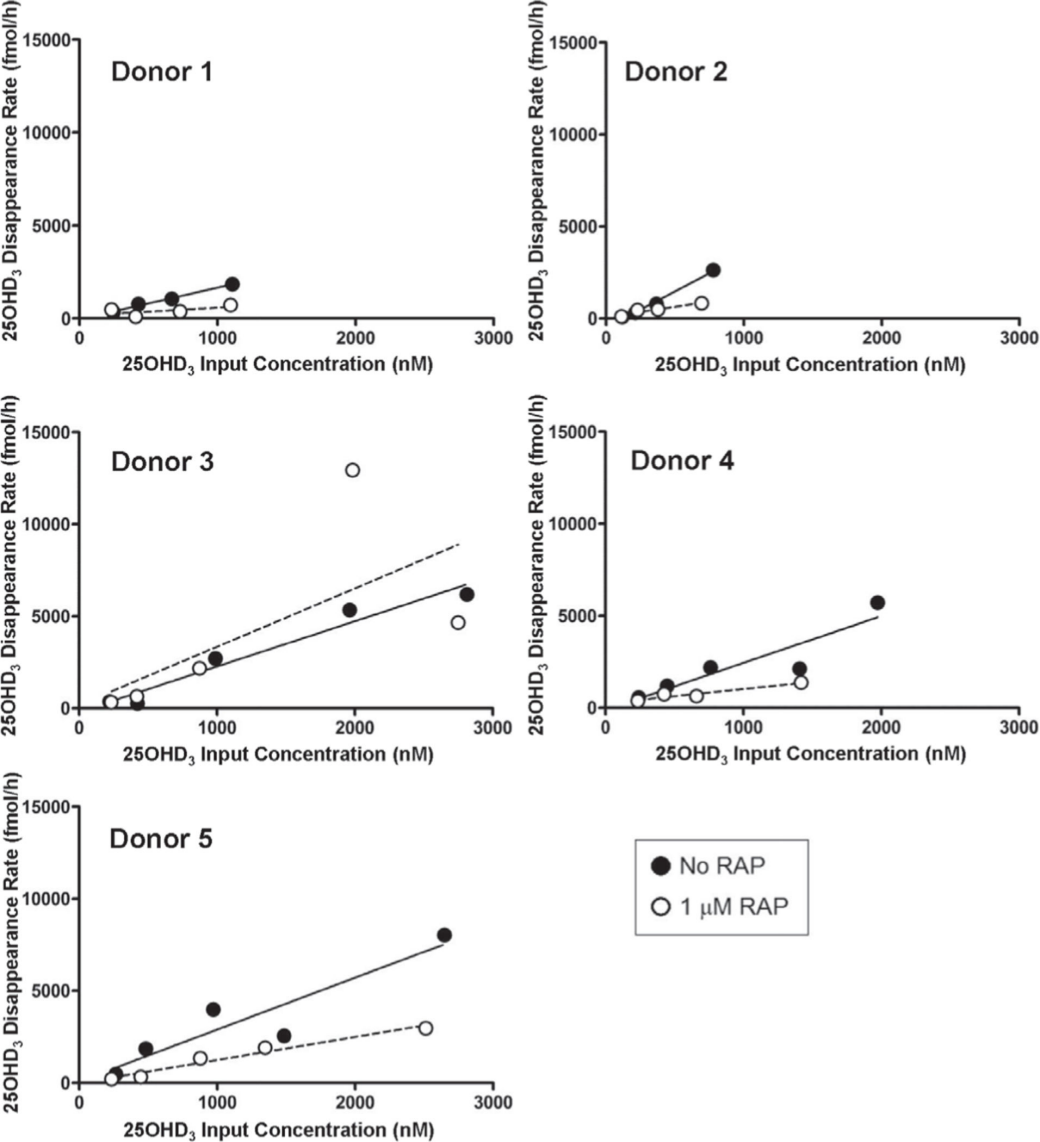
Megalin-mediated cellular uptake and loss of DBP-bound 25OHD_3_ in the PT-MPS The rate of loss of 25OHD_3_ from the perfusion medium was observed to be roughly proportional to the 25OHD_3_ input concentrations across the measured range of input concentrations tested. RAP significantly reduced the estimated intrinsic clearance, reflected in the slope of the linear regression line, of 25OHD_3_ from the luminal perfusion medium.

PT-MPS were perfused with 25OHD_3_ ± RAP. Both the rate of 24,25(OH)_2_D_3_ formation (Fig. 4) and the loss of 25OHD_3_ (Fig. 5) from the perfusion medium increased proportionally with measured 25OHD_3_ input concentrations. Co-administration of RAP resulted in a significant 2.4-fold reduction in the intrinsic clearance of 25OHD_3_ (*CL*_*int,25OHD3*_) from the perfusion medium, confirming a role of megalin in the uptake of DBP-bound 25OHD_3_ in human PTECs. Because 1 μM RAP fully, but selectively, inhibits binding of DBP to megalin and the megalin-dependent co-receptor cubilin (Niemeier et al., 1999; Rowling et al., 2006), other non-receptor mediated endocytotic processes, as well as passive diffusion across the PTEC apical membranes, may account for the residual uptake of 25OHD_3_ into the PTECs observed during RAP co-administration. The experiment was not designed to specifically identify the relative contributions of these residual uptake processes. Finally, megalin inhibition had no effect on the appearance of 24,25(OH)_2_D_3_ in the perfusion medium (Tab. 1), suggesting the intracellular fate of endocytosed 25OHD_3_ to be complex.

### 3.4 Megalin inhibition impairs 1α,25(OH)_2_D_3_-mediated induction of CYP24A1

**Fig. 6:**
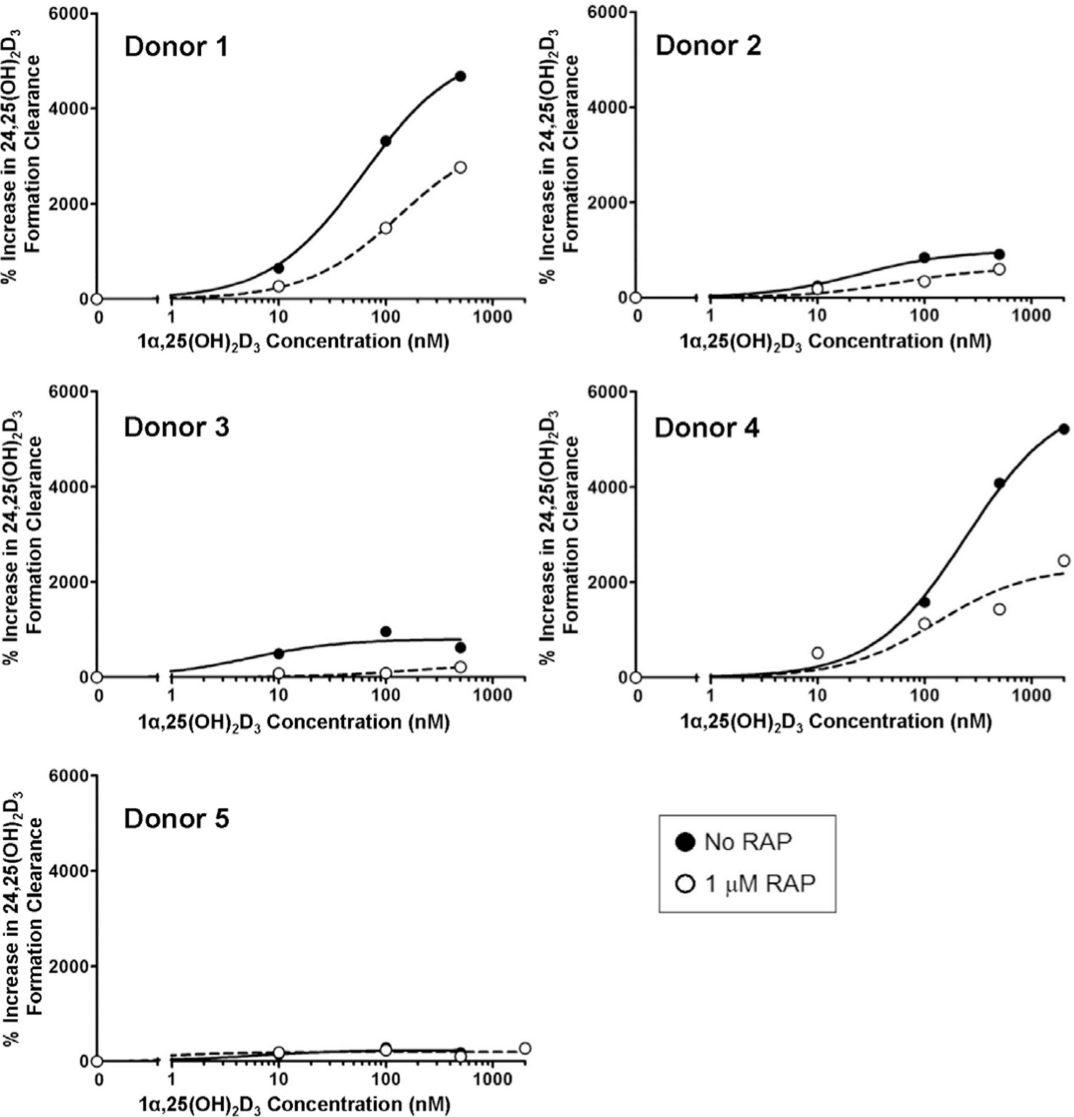
Megalin-mediated uptake of DBP-bound 1α,25(OH)_2_D_3_ is critical for maximal induction of CYP24A1 activity in the PT-MPS The formation of 24,25(OH)_2_D_3_ from 25OHD_3_ increased with increasing concentrations of 1α,25(OH)_2_D_3_. Co-incubation with RAP resulted in a statistically significant downward shift in the maximal inductive response (*E*_*max*_) but had no significant effect on the concentration required for half-maximal effect (*EC*_*50*_).

**Fig. 7:**
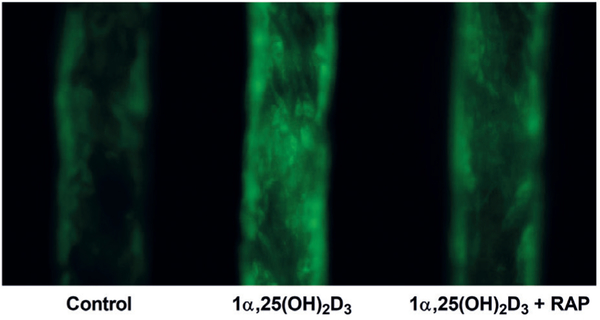
Megalin inhibition impairs 1α,25(OH)_2_D_3_mediated induction of CYP24A1 protein accumulation in the PT-MPS Treatment of PTECs in the PT-MPS with 500 nM 1α,25(OH)_2_D_3_ (center) resulted in increases in fluorescent signal for CYP24A1 protein compared to the vehicle control (left). This inductive effect appeared to be partially reversed with RAP coadministration (right). For reference, the approximate diameter of PT-MPS tubules shown above is 120 μm.

Bioactive 1α,25(OH)_2_D_3_ circulates tightly bound to DBP (Bikle et al., 1985), but the role of megalin in its delivery to the renal proximal tubule has thus far not been evaluated. We assessed whether megalin-mediated uptake of DBP-bound 1α,25(OH)_2_D_3_ modulates the intracellular availability of 1α,25(OH)_2_D_3_ for VDR-dependent biological activity (e.g., induction of *CYP24A1*-mediated 24-hydroxylation of 25OHD_3_). First, PT-MPS were perfused with 25OHD_3_ and a baseline value of 24,25(OH)_2_D_3_ formation clearance was determined. Then, varying concentrations of 1α,25(OH)_2_D_3_ ± RAP were added to the perfusion medium and the increase in the formation of 24,25(OH)_2_D_3_ was calculated. The mean 1α,25(OH)_2_D_3_ concentration required for half-maximal (*EC*_*50*_) induction of 24,25(OH)_2_D_3_ formation displayed a non-significant 1.5-fold rightward shift upon RAP co-treatment (Fig. 6). More importantly, inhibition of megalin resulted in a significant 1.8-fold downward shift in the *E*_*max*_ (Tab. 2). This phenomenon was explained by the partial reversal of the 1α,25(OH)_2_D_3_-mediated increase in CYP24A1 protein levels upon RAP co-administration (Fig. 7). The extent of reversibility was moderate but consistent with the analogous moderate reductions in the enzymatic activity data. Given the nature of RAP as an inhibitor of megalin-mediated processes, the presence of other non-receptor mediated endocytotic processes and passive diffusion across the PTEC apical membranes may account for the incomplete suppression of 1α,25(OH)_2_D_3_-mediated induction of *CYP24A1* observed during RAP co-administration. While the endpoint for the experiment was fold-induction in the 24-hydroxylation of 25OHD_3_, a similar fold-increase in 24-hydroxylation of 1α,25(OH)_2_D_3_ would be expected given that *CYP24A1* is the only enzyme capable of catalyzing hydroxylation at the 24 position for either substrate and the 1α,25(OH)_2_D_3_-mediated induction of enzymatic activity would be proportional to increases in CYP24A1 protein expression for either substrate.

### 3.5 1α,25(OH)_2_D_3_ suppresses megalin gene expression in PTECs

**Fig. 8:**
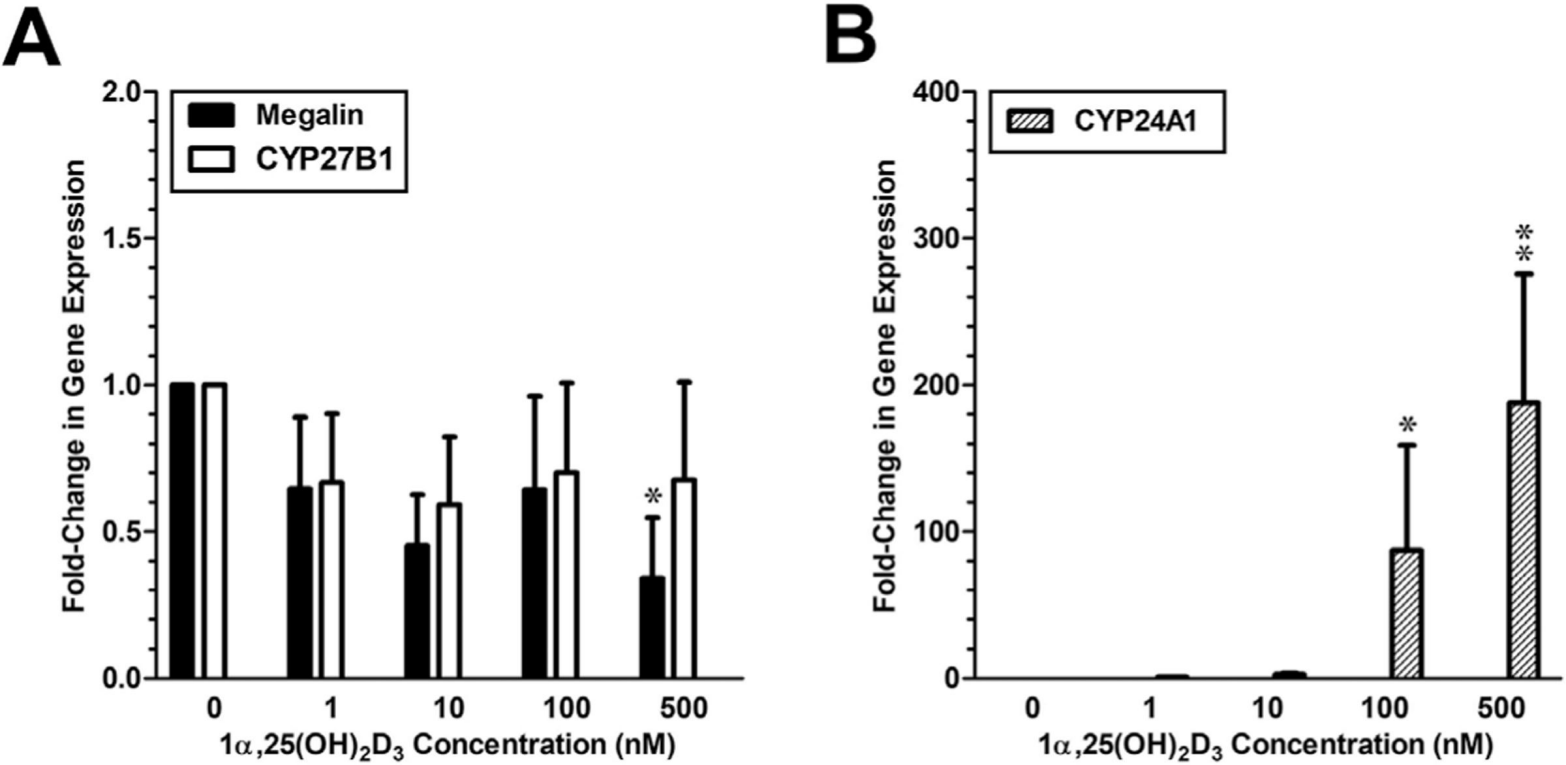
Comparative effects of 1α,25(OH)_2_D_3_ on *megalin*, *CYP24A1* and *CYP27B1* gene expression in PTECs (A) Expression of both *megalin* and *CYP27B1* mRNA expression was suppressed in a roughly dosedependent manner by 1α,25(OH)_2_D_3_. The apparent suppression was statistically significant only for *megalin* and only at the 500 nM dose. (B) Upregulation of the VDR-responsive “inductive” control gene (*CYP24A1*) was observed at both the 100 nM and 500 nM doses of 1α,25(OH)_2_D_3_. Levels of *CYP24A1* RNA were below the limit of quantification in the vehicle control group so the group was omitted from the statistical analysis. All gene expression data was standardized to GAPDH. *, p < 0.05; **, p < 0.01.

We investigated whether megalin gene expression in human PTECs is regulated by 1α,25(OH)_2_D_3_, as has been observed in rat PTECs (Liu et al., 1998). As shown in Figure 8A, both megalin and *CYP27B1* mRNA were not induced by 1α,25(OH)_2_D_3_. Instead, a one-way ANOVA revealed a significant trend of suppression of megalin gene expression by 1α,25(OH)_2_D_3_. Post-hoc ratio t-tests revealed a significant difference between megalin gene expression between the control and 1α,25(OH)_2_D_3_ at the highest (500 nM) dose. Significant up-regulation of the VDR-responsive “inductive” control gene (*CYP24A1*) was also present (Fig. 8B).

## 4 Discussion

In this study, we observed several key findings: (1) megalin plays a critical role in the delivery of DBP-bound 25OHD_3_ to the human proximal tubule, (2) megalin-mediated endocytosis of DBP-bound 1α,25(OH)_2_D_3_ is essential to achieve the maximal physiological response, and (3) elevated 1α,25(OH)_2_D_3_ levels do not induce, and may even suppress, megalin gene expression in human PTECs, contrasting with previously published observations of induction in immortalized rat PTECs (Liu et al., 1998). These results show for the first time in a human cell-derived system the importance of megalin in the uptake of 25OHD_3_ from glomerular ultrafiltrate and extend that critical function to include the uptake and cellular response to 1α,25(OH)_2_D_3_.

Like 25OHD_3_, 1α,25(OH)_2_D_3_ circulates in the blood tightly bound to DBP (Bikle et al., 1985). Thus, megalin-mediated uptake of DBP-bound 1α,25(OH)_2_D_3_ may permit PTECs to better sense and respond to changes in circulating concentrations of this hormone (Bikle et al., 1985). The effects of megalin inhibition on 1α,25(OH)_2_D_3_ hormonal activity manifested predominantly as a reduction in the *E*_*max*_ for *CYP24A1* induction, underscoring the importance of DBP in the intracellular trafficking pathways by which endocytosed megalin transfers DBP-bound 1α,25(OH)_2_D_3_ to the intracellular vitamin D binding proteins (IDBPs) critical in achieving maximal induction of *CYP24A1*. These conclusions are bolstered by previous observations of direct interactions between IDBPs and megalin (Adams et al., 2003). In particular, IDBP-1 has been shown to bind 1α,25(OH)_2_D_3_ with a high capacity and to translocate to the nucleus where it can facilitate the delivery of bound 1α,25(OH)_2_D_3_ to VDR (Gacad and Adams, 1993, 1998; Adams et al., 2007). Our observations form the basis upon which further trafficking studies can be designed to identify the specific proteins in the megalin-initiated chain of custody that carries the different vitamin D metabolites to their specific intracellular targets.

**Fig. 9:**
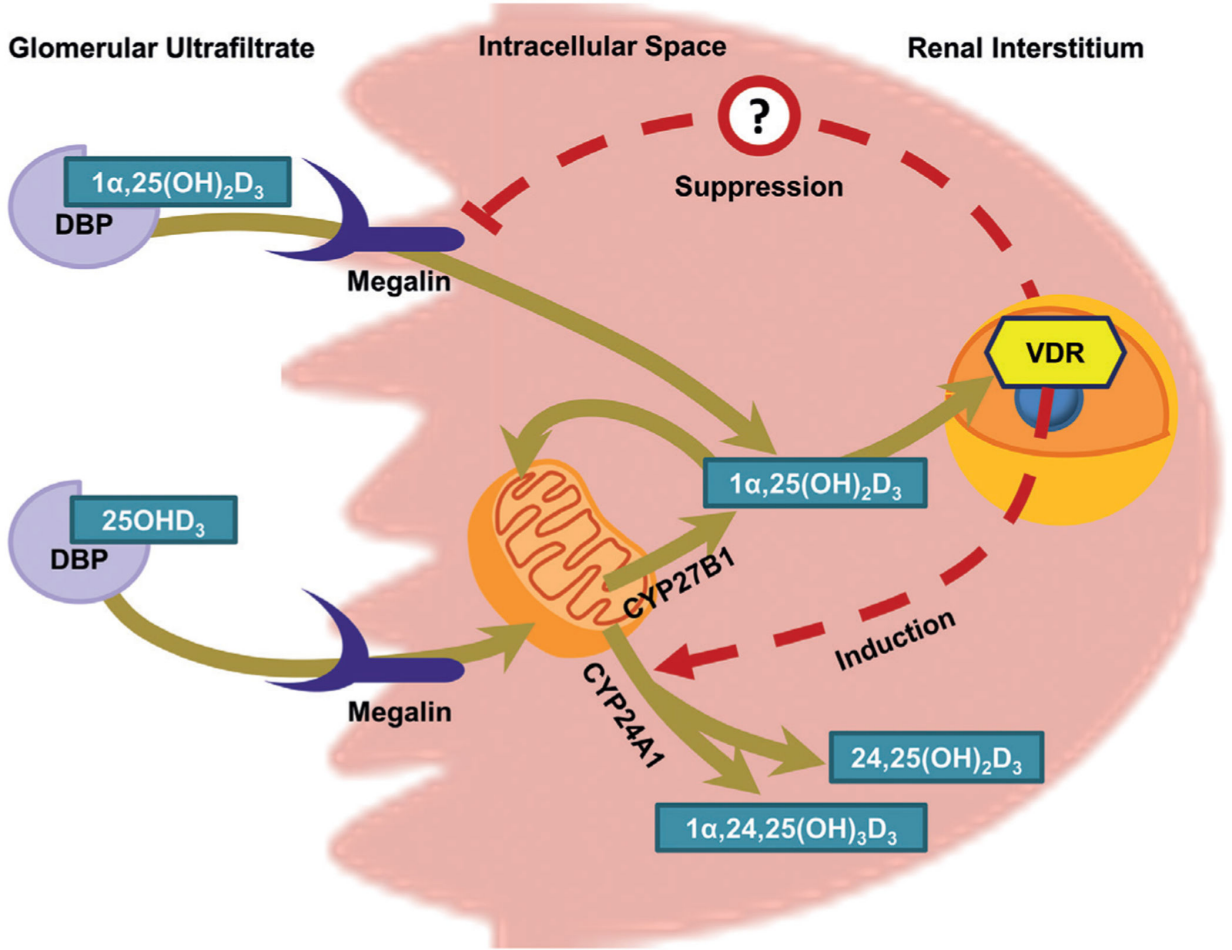
Role of *megalin* in the maintenance of renal vitamin D metabolite homeostasis In PTECs, apically-localized megalin reclaims DBP-bound vitamin D metabolites from the glomerular ultra-filtrate. Resorbed 25OHD_3_ is then shuttled to the mitochondria for either CYP24A1-mediated inactivation or CYP27B1-mediated bioactivation. Intracellular 1α,25(OH)_2_D_3_, whether generated within the PTECs mitochondria or resorbed directly from the tubular lumen, can then undergo metabolic inactivation, enter systemic circulation to mediate endocrine effects or exert VDR-dependent intracrine effects within the PTECs. Intracrine effects include the compensatory upregulation of *CYP24A1* gene expression. *Megalin* gene expression may also be a target for 1α,25(OH)_2_D_3_,- mediated suppression in humans.

Taken together, our findings reveal a complex interplay between DBP levels, megalin-mediated uptake, vitamin D bioactivation and the 1α,25(OH)_2_D_3_-mediated induction of its own metabolic inactivation. In order to characterize further homeostatic aspects of this interplay, we investigated whether 1α,25(OH)_2_D_3_ participates in the reciprocal regulation on megalin gene expression. Hereto, the body of literature evaluating the role of 1α,25(OH)_2_D_3_ in megalin regulation has consisted of a sole study in immortalized rat PTECs, which concluded that 1α,25(OH)_2_D_3_ induces the expression of megalin in the human proximal tubule (Liu et al., 1998). The findings of that singular study have served as the basis for a “vicious cycle” hypothesis (Dusso et al., 2011; Dusso, 2011; Kim and Kim, 2014), which states that CKD-related decreases in renal vitamin D bioactivation can lead to reductions in renal megalin expression (Bosworth and de Boer, 2013), reduce 25OHD_3_ uptake and biotransformation, and result in a cycle of progressively worsening vitamin D deficiency. Our findings are at odds with this hypothesis and even suggest a hypothesis of compensatory negative feedback, whereby PTECs would compensate for diminishing renal vitamin D bioactivation by increasing megalin and its resorptive uptake of DBP-bound 25OHD_3_ into PTECs (Fig. 9). However, with pairwise comparisons, the suppression of megalin gene expression was statistically significant at only 500 nM 1α,25(OH)_2_D_3_, a superphysiological concentration. However, the previous experiments with immortalized rat PTECs, upon which the “vicious cycle” hypothesis is predicated, were conducted at an even higher (1000 nM) concentration (Liu et al., 1998). These findings call into question the regulation of megalin in the kidney by 1α,25(OH)_2_D_3_ *in vivo*. That said, negative feedback loops have consistently characterized the physiological regulation of systemic vitamin D homeostasis (e.g., induction of *CYP24A1* and CYP27B1 by 1α,25(OH)_2_D_3_ and parathyroid hormone, respectively) (Jones et al., 2012; Dusso et al., 2005; Brenza et al., 1998). Our findings are more in line with this overarching theme of compensatory regulation and reconcile published results that previously seemed anomalous, such as the down-regulation of megalin gene expression in LLC-PK1 cells exposed to the VDR ligand, lithocholic acid (Erranz et al., 2004).

In summary, our findings describe megalin as a protein intimately woven into the complex web of physiological mechanisms regulating mineral homeostasis by promoting the uptake of 25OHD_3_ and 1α,25(OH)_2_D_3_ into human PTECs. Further elucidation of aspects of this regulatory process may provide a better understanding of renal physiology and possibly reveal novel therapeutic targets for CKD. While megalin-knockout mice and rodent cell systems have been useful in the initial identification of megalin as an important regulator of vitamin D homeostasis, concurrent advances in primary human cell culture techniques and microphysiological cell culture platforms now offer promising alternatives to traditional non-human animal models for exploring the many further questions relating to renal vitamin D homeostasis and other important organ functions.

## Supplementary Material

Supplemental Information

## Figures and Tables

**Tab. 1:** Donor-specific effects of RAP on the intrinsic clearances of 24,25(OH))_2_D_3_ formation and 25OHD_3_ loss Treatment with the megalin inhibitor (RAP) significantly reduced the intrinsic clearance for 25OHD_3_ loss (*CL*_*int,25OHD3*_) to PTECs cultured in the PT-MPS but had no significant effect on the intrinsic clearance of 24,25(OH)_2_D_3_ formation (CL_*int,24,25(OH)2D3*_).

	*CL*_*int,24,25(OH)2D3*_ (μl/h)			*CL*_*int,25OHD3*_ (μl/h)		
Donor No.	No RAP	RAP	Fold-change *CL*_*int*_	No RAP	RAP	Fold-change *CL*_*int*_
1	0.015	0.021	1.4 ↑	1.7	0.42	4.0 ↓
2	0.00029	0.00037	1.3 ↑	4.0	1.1	3.6 ↓
3	0.038	0.036	1.1 ↓	2.5	3.2	1.3 ↑
4	0.0052	0.0021	2.5 ↓	2.6	0.78	3.3 ↓
5	0.056	0.052	1.1 ↓	2.8	1.3	2.2 ↓
Mean^†^	0.023	0.022	1.1 ↓	2.7	1.4	*2.4 ↓
SD^†^	0.024	0.022	1.6	0.83	1.1	2.0

† Geometric mean and geometric standard deviation calculated for “fold-change” (ratio) parameters.

* , p < 0.05.

**Tab. 2:** Donor-specific effects of RAP on 1α,25(OH)_2_D_3_-mediated induction of 24,25(OH)_2_D_3_ formation clearance Treatment with the megalin inhibitor (RAP) significantly reduced the maximal inducibility (*E*_*max*_) for the 1α,25(OH)_2_D_3_-mediated induction of 24,25(OH)_2_D_3_ formation clearance in the PT-MPS but had no significant effect on the concentration of 1α,25(OH)_2_D_3_ required for halfmaximal induction (*EC*_*50*_) of 24,25(OH)_2_D_3_ formation clearance.

	*EC*_*50*_ (nM)			*E*_*max*_ (% Increase from baseline)		
Donor No.	No RAP	RAP	Fold-change *EC_50_*	No RAP	RAP	Fold-change *E_max_*
1	62	130	2.1 ↑	5300	3500	1.5 ↓
2	25	56	2.2 ↑	990	640	1.5 ↓
3	5.3	170	32 ↑	800	280	2.9 ↓
4	240	130	1.8 ↓	5900	2300	2.6 ↓
5	5.6	0.61	9.2 ↓	235	200	1.2 ↓
Mean^†^	68	97	1.5 ↑	2600	1400	*1.8 ↓
SD^†^	99	68	8.1	2700	1500	1.5

† Geometric mean and geometric standard deviation calculated for “fold-change” (ratio) parameters.

* , p < 0.05.

## Abbreviations

1α,25(OH)_2_D_3_: 1α,25-dihydroxyvitamin D_3_
24,25(OH)_2_D_3_: 24,25-dihydroxyvitamin D_3_
25OHD_3_: 25-hydroxyvitamin D_3_
CKD: chronic kidney disease
CYP: cytochrome P450
DBP: vitamin D binding protein
***EC***_*50*_: concentration of inducer at which half-maximal effect occurs
***E***_*max*_: predicted maximal inductive effect
FBS: fetal bovine serum
PTECs: proximal tubule epithelial cells
PT-MPS: proximal tubule microphysiological system
RAP: receptor-associated protein
VDR: vitamin D receptor
