# Supplementary material for: Reevaluating the Role of Megalin in Renal Vitamin D Homeostasis Using a Human Cell-Derived Microphysiological System
Source: ALTEX. Author manuscript; Available in PMC 2019 Dec 6. (PMC6896899; doi:10.14573/altex.1803161)
Supplement: Supplemental Information [file NIHMS1048695-supplement-Supplemental_Information.pdf]

Chapron et al.:

Reevaluating the Role of Megalin in Renal Vitamin D Homeostasis Using a Human Cell-Derived Microphysiological System

Supplementary Data

Supplementary materials

Validation of DBP as a delivery vehicle for vitamin D metabolites in the PT-MPS

After 5 days under standard culture conditions, 1 µM $25OHD_3$ was added to the PT-MPS media perfusate with either the previously established delivery vehicle, 2% FBS,(Weber et al., 2016) or the experimental delivery vehicle, 3 µM purified human DBP. Media exiting the PT-MPS luminal path was collected over 24-hour intervals, for 3 days. All samples were stored at -80°C until they could be prepared for LC/MS-MS analysis using a previously established method (Weber et al., 2016, Wang et al., 2011). Quadruplicate PT-MPS (technical replicates) were used for each of the kidney tissue donors evaluated (biological replicates, n = 3). Some $24,25(OH)_2D_3$ contaminant was present in both the FBS and the plasma-derived human DBP. As such, net appearance rate, rather absolute appearance rate of $24,25(OH)_2D_3$ in the efflux media was used for the assessment of vitamin metabolite disposition in the PT-MPS (Equation S1).

$$Net\ Efflux\ Appearance\ Rate_{24,25(OH)_2D_3} = \frac{[24,25(OH)_2D_3]_{outflow} - [24,25(OH)_2D_3]_{input}}{Duration\ of\ Collection\ Interval\ /\ Volume\ Collected} \qquad \text{(S1)}$$

The mean and standard deviation of net efflux appearance rate were calculated for each treatment (DBP or FBS) at each time point and two-way ANOVA was performed. A post-hoc Bonferroni procedure was used to make pairwise comparisons. Results are presented in Figure S1.

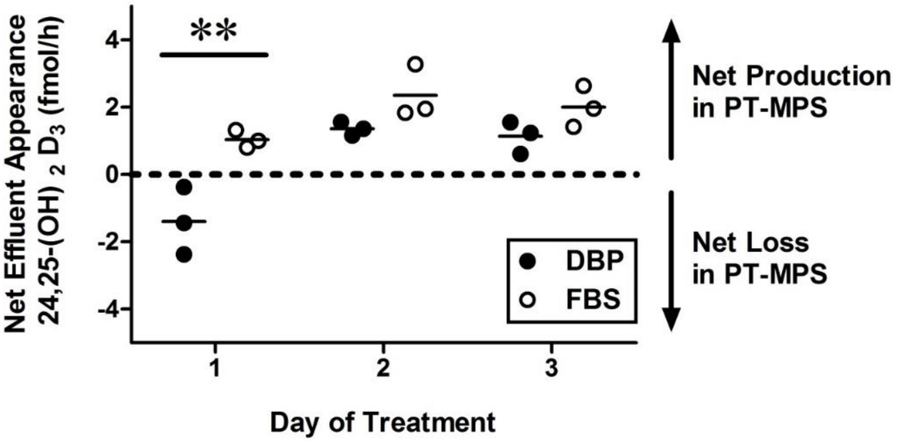

Fig. S1: Validation of DBP as a carrier protein for vitamin D metabolites in the PT-MPS

Cells cultured in the PT-MPS with either 2% FBS or 3 µM DBP as delivery vehicle for vitamin D metabolites exhibited net $24,25(OH)_2D_3$ formation during the 24-48 h and 48-72 h collection intervals. Unlike with FBS, cells cultured with DBP exhibited a net loss of $24,25(OH)_2D_3$ to the PT-MPS system during the 0-24 h collection interval. The difference in net $24,25(OH)_2D_3$ formation rate between the FBS- and DBP-treated cells was significant for the 0-24 h collection interval. **Very statistically significant, $p < 0.01$. These results demonstrate that purified human DBP (which provides a more controlled culture milieu than 2% v/v FBS) is an effective vehicle for the delivery of vitamin D metabolites to the PT-MPS.

doi:10.14573/altex.1803161s

Tab. S1: Antibody information

RRID	Protein target	Antigen sequence	Name of antibody	Manufacturer, Catalog No.	Species raised in, monoclonal or polyclonal	Dilution used
AB_2640319	Recombinant protein corresponding to human CYP24A1	unknown	CYP24A1 Polyclonal Antibody	Thermo-Fisher, PA5-54579	Goat, polyclonal	1:200
AB_10673466	Synthetic peptide within human megalin 4600 to C-terminus	unknown	Anti-Lrp2 / Megalin Antibody	Abcam, ab76969	Rabbit, polyclonal	1:25
AB_306023	Full length native purified protein rabbit alpha 1 sodium potassium ATPase	unknown	Anti-alpha 1 Sodium Potassium ATPase Antibody	Abcam, ab7671	Mouse, monoclonal	1:100

Tab. S2: Kidney tissue donor characteristics in 25OHD$_3$ uptake/loss experiment

Donor No.	Age	Sex	Ethnicity	Pre-existing conditions	Reason for nephrectomy	Final pathology
1	58	Male	White	HTN, sleep apnea	Renal mass (bilateral)	Papillary RCC, multiple papillary adenomas
2	56	Male	White	None reported	Renal mass (left)	Clear cell RCC
3	58	Male	White	HTN, hernia	Renal mass (right)	Unclassified RCC with clear cell features
4	58	Male	White	None reported	Renal mass (right)	Clear cell RCC
5	40	Male	African American	Methamphetamine use, deep vein thrombosis	Renal mass (left)	Clear Cell RCC

Tab. S3: Kidney tissue donor characteristics in 1α,25(OH)$_2$D$_3$-mediated induction experiment

Donor No.	Age	Sex	Ethnicity	Pre-existing conditions	Reason for nephrectomy	Final pathology
1	65	Male	White	None reported	Renal mass	Not available
2	49	Male	White	Clear cell RCC	Renal mass	Not available
3	68	Male	White	HTN, GERD, Aortic Aneurysm, Diabetes Mellitus 2	Renal mass (left)	Clear cell RCC
4	62	Female	White	HTN, GERD, previous cervical, thyroid and breast carcinoma	Renal mass	Not available
5	58	Male	White	HTN, sleep apnea	Renal mass (bilateral)	Papillary RCC, multiple papillary adenomas
